# 

**Published:** 1871-04

**Authors:** 


### CONTENTS:

### ORIGINAL COMMUNICATIONS.

ARTICLE I.—Inflammation of the Dental Pulp. (From the German of Prof. C. Wedl.) By Jas. H. Ludwig, M.D., D.D.S. .......... 529
ARTICLE II.—Professional Shortcomings. By J. H. Foster, M.D. .......... 540
ARTICLE III.—Disease of the Antrum. By Edward W. Anderson, D.D.S. .......... 550

### SELECTED ARTICLES.

ARTICLE IV.—"The Patent Flexible Edge." By W. Geo. Beers, L.D.S. .......... 553

ARTICLE V.—Poisoning from Chloral Hydrate .......... 556

ARTICLE VI.—Case of Tumor Originating in the Fauces. By Kelburne King, M.D., F.R.C.S. .......... 559

ARTICLE VII.—Indications for Lancing the Gums .......... 565

### MONTHLY SUMMARY.

Hypertrophy of the Cementum—Exostosis .......... 567

Function of the Prostate Gland .......... 569

Fibro-Serous Cyst of Jaw .......... 569

Neuralgia of Jaw .......... 570

The Virtue of the Sunflower in Intermittent Fever .......... 571

Test for the Purity of Chloral .......... 571

Undeveloped Eye Tooth .......... 572

Osborn's Neuralgic Pill .......... 572

### EDITORIAL DEPARTMENT.

Dental Items. "An Improved Method of Filling Teeth." .......... 572
Inflammability of the Paroxyline Base .......... 573
Method of Casting Aluminum Plates .......... 573
Impressions of Difficult Partial Cases .......... 575
Erratum. Dr. Geo. B. Harriman .......... 576
Index to Volume 4.—Third Series.

